# A Case of Metastatic Basal Cell Carcinoma (BCC) With Spinal and Pulmonary Metastases Treated With Vismodegib, Sonedigib, and Radiotherapy

**DOI:** 10.7759/cureus.23273

**Published:** 2022-03-17

**Authors:** Arthur M Samia, Joseph M Nenow, Philip Boyer

**Affiliations:** 1 Dermatology, University of Florida, Gainesville, USA; 2 Internal Medicine, Indiana University, Indianapolis, USA; 3 Department of Pathology and Laboratory Medicine, Vidant Medical Center/East Carolina University, Greenville, USA

**Keywords:** spine, pathology, metastases, lung, dermatology, basal cell carcinoma

## Abstract

Basal cell carcinoma (BCC) is the most common malignancy worldwide and has one of the most favorable prognoses due to its tendency to remain local. Clinical presentation with rare distant metastases significantly increases morbidity and mortality. Historically, no effective therapies have existed for locally advanced or metastatic BCC. Recent research highlights the possibility of treating patients with advanced and metastatic BCC with hedgehog pathway inhibitors, such as vismodegib or sonedigib. We present the case of a 62-year-old male with a history of a large left shoulder lesion, which was diagnosed as a nodulocystic BCC following biopsy and histopathologic examination. The primary lesion was managed with surgical excision, and his ensuing metastatic disease was treated with vismodegib, sonedigib, tumor debulking, and radiation therapy. Magnetic resonance imaging and computed tomography of the chest revealed probable metastases to the apical segment of the left upper lobe and thoracic spine, leading to spinal stenosis and probable cause of the patient's ataxia and paresthesias. Due to the ability of BCCs to transform during metastasis, it is impossible to identify the nature of metastatic lesions (i.e., basaloid, squamous, or hybrid) without biopsy. In this case report, we review the etiologies, typical demographics, presentation patterns, and treatment regimens for metastatic BCC and the possibility of metastatic disease transforming to squamous or hybrid variants.

## Introduction

Basal cell carcinoma (BCC) is the most common malignancy worldwide and has a favorable mortality rate due to its tendency to remain local [1]. Clinical presentation with distant metastases is rare and significantly increases morbidity and mortality [1]. Due to metastatic BCC's combination of rarity and the severity of prognosis, it remains difficult yet essential to diagnose early, especially considering the possibility of intervening [2]. When diagnosed early, most BCCs are treated with in-office-based therapies varying from topical agents and superficially destructive methods to more invasive techniques, including surgical procedures [3]. The therapeutic modality employed is typically influenced by the BCC's morphological subtype (e.g., nodular, superficial, morpheaform/infiltrative, basosquamous, pigmented) [3]. These techniques function best with lower-stage cancers as no effective therapy exists for locally advanced or metastatic carcinoma [3]. More recently, emerging molecularly targeted therapies, vismodegib and sonedigib, have shown great potential for use rate among patients with advanced BCC [4,5].

## Case presentation

**Figure 1:**
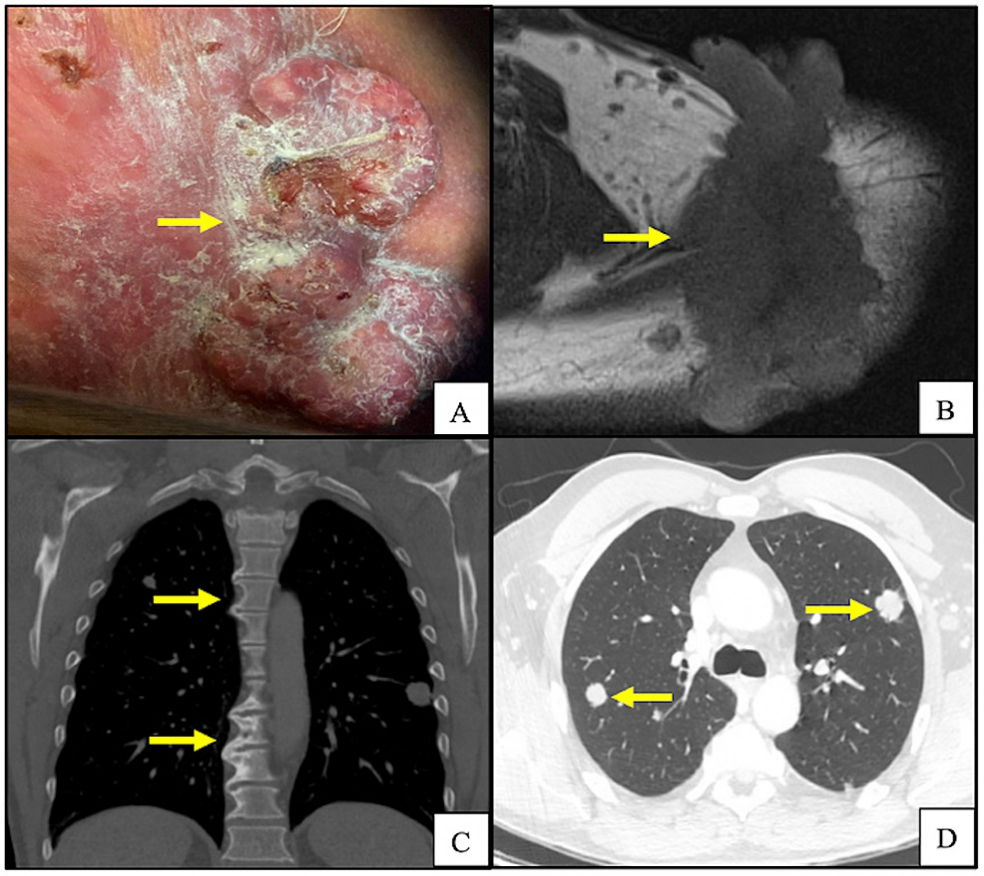
A) Gross pathology of BCC on the left shoulder at the time of diagnosis; B) axial MRI imaging of left shoulder at the time of diagnosis, image is indicative of invasive BCC; C) chest CT scan in bone window demonstrating sclerotic changes at T2/T3 and T6-T8 concerning for bony metastasis; D) chest CT scan in lung window demonstrating bilateral pulmonary nodules found to be metastatic BCC. Yellow arrows indicate abnormal pathology.

**Figure 2:**
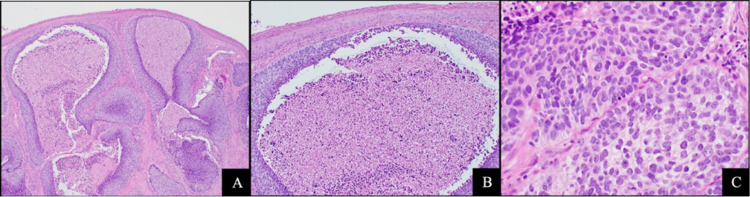
Primary basal cell carcinoma 40x, 100x, and 200x magnification view (from left to right) with H&E staining

**Figure 3:**
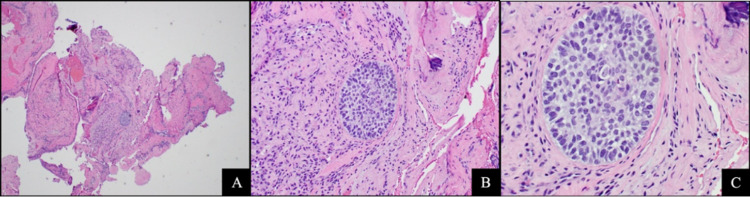
Basal cell carcinoma pulmonary metastasis 40x, 100x, and 200x magnification view (from left to right) with H&E staining

**Figure 4:**
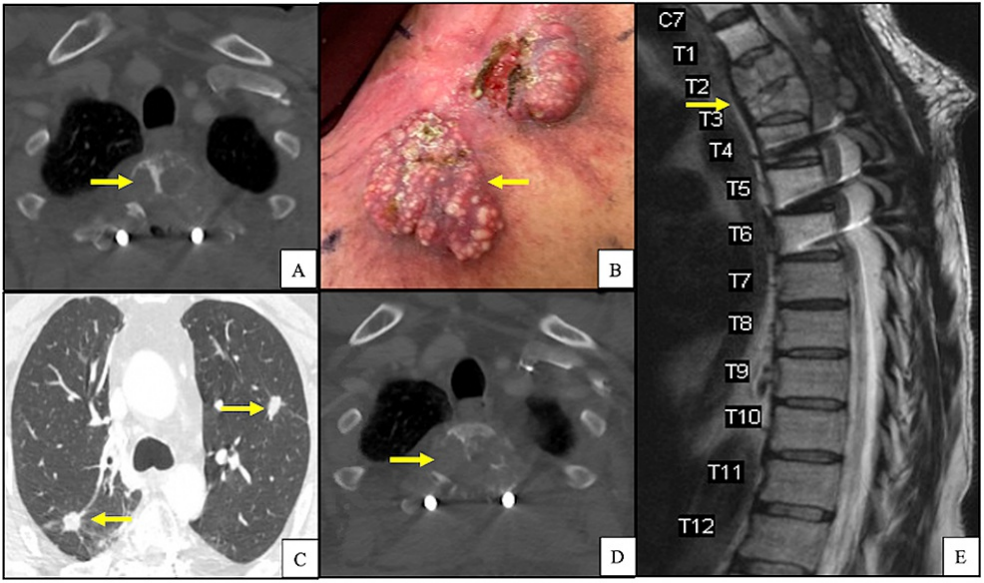
A) Chest CT scan in bone window demonstrating lytic changes at T2 level with histopathologic confirmation of metastatic BCC following radiation and vismodegib therapy; B) gross pathology on the left shoulder of BCC concerning for cutaneous squamous cell carcinoma transformation after secondary hedgehog inhibitor therapy; C) chest CT scan in lung window demonstrating stable bilateral pulmonary nodules 30 months after initial diagnosis; D) chest CT scan in bone window demonstrating progressive lytic changes at T2 level with histopathologic confirmation of metastatic BCC 30 months after initial diagnosis; E) sagittal T2-weighted MRI of spine demonstrating bony metastasis at T2/T3 with thoracic spinal cord compression after presenting with worsening lower extremity weakness. Yellow arrows indicate abnormal pathology.

A 62-year-old Caucasian male presented to our institution with a fungating left shoulder mass (Figure 1A). The patient reported that he first noticed the mass three years prior and had not sought medical attention. Further workup and imaging revealed primary tumor invasion into the left lower neck with lymphadenopathy along with pulmonary and osseous metastases (Figures 1B-1D). Histopathologic examination of the primary lesion showed an atypical basaloid proliferation with peripheral palisading architecture extending into a fibrotic and inflamed dermis (Figures 2A-2C). Histopathologic examination of pulmonary lesions confirmed BCC metastases (Figures 3A-3C). Tumor cells were positive for cytokeratin (CK) 5, CK 6, and tumor protein p63 but negative for carcinoembryonic antigen and epithelial membrane antigen. He was staged at Stage IV (T2, NX, M1) based on the American Joint Committee on Cancer 7th edition guidelines. The patient was promptly treated with vismodegib and had interval improvement in pulmonary and osseous metastatic disease. One year later, the patient presented to the emergency department with new back pain and ataxia, he was found to have a new lytic lesion at T2/T3 (Figure 4A), and the patient underwent cervicothoracic decompression, fusion reduction, and T2 and T3 laminectomies. The accompanying bone biopsy confirmed BCC metastasis. The patient was hospitalized for rehabilitation following a neurosurgical intervention. The patient completed palliative radiation treatment for metastatic spinal disease and was started on sonedigib for disease progression. A serial biopsy of a metastatic lesion was concerning for BCC to cutaneous squamous cell carcinoma transformation (Figure 4B). Interval imaging over the following 18 months noted the patient to have a mild progression of thoracic spine disease with stable pulmonary disease (Figures 4C-4D). At the time, he denied any bowel or bladder incontinence but had been stumbling due to increasing leg weakness. Physical examination upon admission noted bilateral lower extremity weakness and paresthesias. MRI showed a tumor compressing the spinal cord (Figure 4E). He underwent thoracic spine debulking procedures and regained mobility prior to discharge. Histopathology again confirmed metastatic BCC.

**Figure 5:**
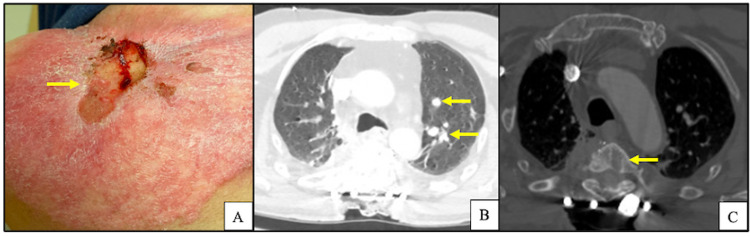
Gross pathology of BCC on the left shoulder at four-year follow-up; B) chest CT scan in lung window demonstrating stable to mildly progressed pulmonary nodules 48 months after initial diagnosis; C) chest CT scan in bone window demonstrating lytic changes at T2/T3 level 48 months after initial diagnosis. Yellow arrows indicate abnormal pathology.

Because of his disease progression, molecular profile analysis was performed and was significant for tumor mutation burden (TMB) high, PD-L1 0%, and mutations in APC, MYCN, NF2, BRCA1, TP53. He was started on pembrolizumab for eight cycles, followed by additional thoracic spine radiation. The patient's pulmonary disease has remained stable to mild progression, and his primary skin lesion has significantly reduced in size (Figures 5A-5B). The patient continues to have neurosurgical care for thoracic cord compression from metastatic spinal disease and has interval imaging performed to monitor his disease (Figure 5C).

## Discussion

Metastatic BCC is most prevalent in males older than 60, with a history of sun exposure or previous radiation treatment in early adulthood [6]. Additionally, there is an association with incomplete local excision of the primary tumor and later progression to metastatic disease [6,7]. The primary tumor is, on average, noted at 45 years of age with a nine-year interval between the tumor's onset and the diagnosis of metastasis [6,7]. Metastasis spread occurs in 1/1,000 and 1/35,000 cases with equally lymphogenic and hematogenic spread to lymph nodes, lungs, and bones [6,7]. Age and sex are unrelated to metastatic spread and survival [6,7]. The median survival time between diagnosis and starting targeted hedgehog pathway inhibitors is eight months to 1.6 years [6,7]. Patients typically present clinically when metastases begin compressing the spinal cord and lead to features of neurologic weakness and sensory abnormalities, as seen in this patient [6,7]. Additionally, pulmonary metastases can clinically present as shortness of breath, dry cough, or hemoptysis [6,7]. Lymph node involvement will typically produce unilateral, non-tender firmness in regional lymph nodes [6,7]. Therefore, a physical examination including neurologic, pulmonary, and lymph node investigations may assist in localizing metastases; however, this is likely impractical in most clinical settings.

In cases of advanced and metastatic BCCs, it is possible to discover concurrent histopathologic evidence of squamous cell carcinoma [8]. The etiologies of this manifestation include collision tumors involving basal and squamous cell carcinomas, an element of squamous cell carcinoma that has independently metastasized, or an instance of a pure BCC that has undergone squamous cell transformation [8]. Although squamous transformation remains a possibility, squamous differentiation in primary lesions was reported in less than 15% of primary tumors and metastases [8].

Vismodegib was chosen for treatment in this patient's case due to its documented effectiveness in a systematic review. Jacobsen et al. noted vismodegib's superior efficacy in treating metastatic cases with responsiveness noted in 34% of cases and complete responsiveness in 4% of cases [5]. By comparison, sonedigib did not qualify for systemic analysis due to limited evidence [5]. Standard therapies such as radiotherapy or surgery should be considered in locally advanced cases [3]. Having mentioned vismodegib's effectiveness, it should be noted that there are reports of BCC to cutaneous squamous cell carcinoma variant transformation following treatment with vismodegib [9-12]. By comparison, in basosquamous subtypes, sonedigib and vismodegib have BCC response rates of 30% and 43%, respectively, in cases of locally invasive and metastatic disease [9]. These treatments are often poorly tolerated given their side effect profiles of gastrointestinal distress, alopecia, and muscle cramping, leading to frequent discontinuation [13].

Since metastatic BCC identification may be challenging and treatment is not frequently tolerated, the best strategy to address metastatic BCCs is prevention. Considering the appreciable number of cases of metastatic BCC associated with incomplete excision followed by immediate wound closure, particularly by grafting, it is important to remember that surgical excision is not always definitively therapeutic in the management of these lesions [14]. Therefore, after large or recurrent BCC excision, wound grafting is recommended to be delayed for at least six months to ensure the tumor was completely excised [14].

## Conclusions

While BCC is the most common malignancy worldwide, it has very low metastatic potential. We presented the case of a 62-year-old Caucasian male with a history of a large, left shoulder BCC status-post excision and treatment with vismodegib, sonedigib, and radiation therapy for metastatic disease. This case serves as an important reminder of BCC's rare metastatic potential and supports the use of tumor debulking, hedgehog pathway inhibitors, and radiotherapy as beneficial therapeutic modalities for metastatic disease. Given the high incidence of BCCs and other cutaneous malignancies, it remains crucial for primary care physicians to urge patients with a significant history of sun exposure or suspicious skin lesions to receive a dermatologic evaluation.
